# Peripheral vascular disease – a new vascular disease associated with normal tension glaucoma: a case report

**DOI:** 10.1186/s13256-020-02533-3

**Published:** 2020-11-19

**Authors:** Ali Nowrouzi, Javier Benitez-del-Castillo, Sepideh Kafi-abasabadi, Mario Rodriguez-Calzadilla, Antonio Diaz-Ramos, Alejo Rodriguez-Suarez, Inmaculada Mota-Chozas

**Affiliations:** Hospital Jerez de la Frontera, Ronda de Circunvalación, Jerez de la Frontera, 11407 Cádiz, Spain

**Keywords:** Normal-tension glaucoma (NTG), Peripheral arterial disease (PAD), Glaucomatous optic neuropathy, Case report

## Abstract

**Introduction:**

Normal-tension glaucoma is known as a multifactorial optic neuropathy. A number of lines of evidence suggested that vascular factors played a significant role in the development of normal-tension glaucoma. The mechanisms underlying the abnormal ocular blood flow in normal-tension glaucoma are still not clear. Peripheral vascular disease seems to be associated with glaucoma populations independent of other cardiovascular risk factors. We found this presentation, for the first time, to our knowledge, as another probable vascular abnormality related to our patient with normal-tension glaucoma, although it is necessary to confirm its pathological effect in future studies.

**Case presentation:**

Our patient was a 48-year-old Spanish man without any personal and family history of interest except for circulatory problems of the lower limbs with repetitive ulcers at the frontal and lateral aspects of his legs. His chief complaint was vision loss when he came to consult us. In exploration, his best corrected visual acuity was 20/20 in both eyes; initial intraocular pressure in the right eye was 14–16 mmHg and in the left eye was 16–18 mmHg, with a mild sclerosis of the lens in slit-lamp examination. No inflammation or pigmented lesion was detected in the anterior chamber. Open angle confirmed by Goldman four quadrants gonioscopy. Funduscopic examination revealed a vertical cup disc ratio of 0.6 in the right eye and 0.8 in the left eye. The patient’s neuroretinal rim was normal in the right eye, and superior thinning in the left eye was determined. Examination of the patient’s visual field showed inferior mild probable nasal scotoma in the right eye and an inferior deep arcuate scotoma defect in the left eye. His optical coherence tomography examination revealed thinning of the peripapillary nerve fiber layer thickness in the left eye and superior loss of macular retinal ganglion cells in the left eye. Normal intraocular pressure values were measured on the intraocular pressure curve without treatment (maximum value, 18–20 mmHg), discarding higher intraocular pressures measured out of office. Ultrasonic pachymetry measured 515/520 μm, and normal intraocular pressure measured with a PASCAL tonometer ruled out probable corneal biomechanical underestimations. The patient’s polysomnography study was normal and excluded sleep apnea syndrome. The patient’s serial mean blood pressure was normal, especially in the lower limbs (mean value, 125/70 mmHg), ruling out the possibility of systemic hypotension. Thyroidal and coagulation abnormalities, autoimmune disease, and inflammatory disease were excluded. Normal immunologic study and normal vascular biopsy were observed, as well as normal brain magnetic resonance imaging and a normal carotid vascular study. The primary diagnosis was moderate medium peripheral arterial disease in the lower limbs, which was confirmed by echography after ruling out other probable vascular abnormalities related to normal-tension glaucoma.

**Conclusion:**

After ruling out other systemic diseases and vascular abnormalities related to normal-tension glaucoma, we found peripheral arterial disease as a probable vascular abnormality related to normal-tension glaucoma in our patient. To our knowledge, this is the first time such a case has been reported. Thus, further research is needed to determine the relevance of these results to the general population.

## Introduction

Normal-tension glaucoma (NTG) is known as a multifactorial optic neuropathy characterized by progressive retinal ganglion cell (RGC) death and glaucomatous visual field loss. Despite the fact that the intraocular pressure (IOP) in patients with NTG is within the normal range, the glaucomatous optic neuropathy may keep getting worse progressively and irreversibly. A significant percentage of patients with NTG (as high as 21%) have a family history of glaucoma and genetic predisposition to the disease. The disturbed ocular blood flow is a significant factor in the pathogenesis of NTG. The vascular failure, including vasospasms, small-vessel disease, or autoregulatory dysfunction, will lead to perfusion deficits of the optic nerve head (ONH) and retina and furthermore develop into glaucomatous optic neuropathy [1]. Previous studies showed that the impaired systemic and ocular vascular autoregulation was more pronounced in NTG than in high-tension glaucoma, especially in patients with NTG with progressive optic neuropathy, than in those with relatively stable status [2, 3]. Therefore, the ocular blood flow deficit may play an important role in the glaucomatous optic neuropathy in NTG.

Systemic vascular diseases, including migraine, systemic low blood pressure (BP), Alzheimer disease, primary vascular dysregulation (PVD), and Flammer syndrome, are associated with the progression of NTG [4]. It is generally accepted that the risk factors for NTG include patient sex [5, 6], patient race (more frequently seen in Asian than in European or American patients) [7], PVD [8], and low BP [9, 10]. Female patients appeared to be more susceptible to vasospasm and progression of visual field loss in NTG [11]. Similarly, vasospasm was more commonly observed in Asian than in European and American patients, and, correspondingly, NTG was more commonly seen in Asian countries than in most other countries [12, 13]. Migraine, a disorder associated with NTG, is characterized as a vasospastic disorder and is commonly seen in women [14].

Peripheral arterial disease (PAD) is a prevalent vascular abnormality. Claudication represents an early yet common manifestation of PAD. A clinical history and physical examination combined with an ankle-brachial index can help make a diagnosis of claudication.

Patients at risk for developing PAD are those who are older than 65 years of age, those with risk factors for atherosclerosis (for example, diabetes mellitus, history of smoking, hyperlipidemia, and hypertension), family history of PAD, and having known atherosclerotic disease in another vascular bed (for example, coronary, carotid). Invasive (angiography) and noninvasive (duplex ultrasound, computed tomographic angiography, or magnetic resonance angiography) anatomic testing of the lower extremities is useful to diagnose anatomic location and severity of stenosis for patients with symptomatic PAD [15, 16].

## Case presentation

**Fig. 1:**
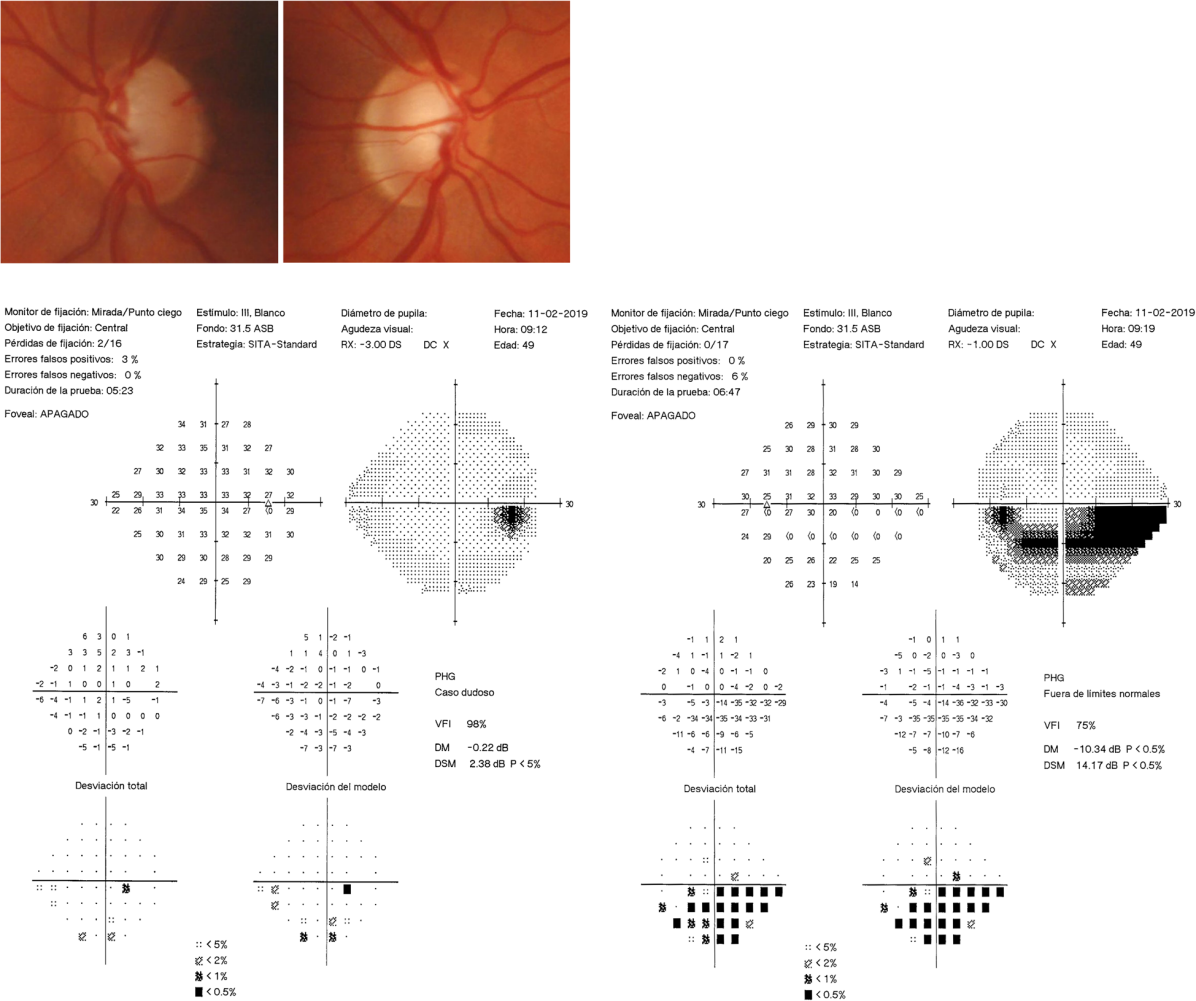
Funduscopic examination and Humphrey visual campimetry

**Fig. 2:**
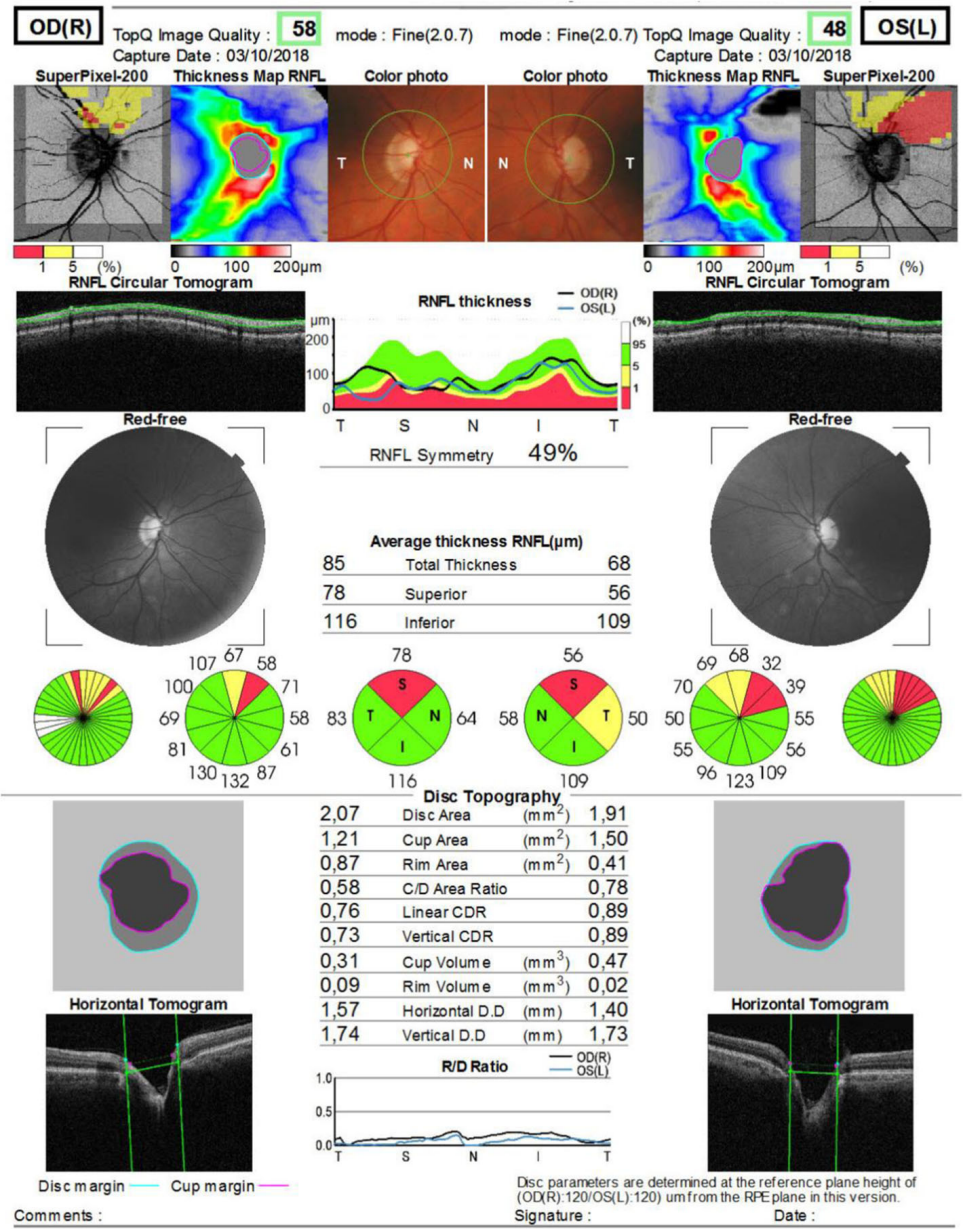
Optical coherence tomography of optic nerves

Our patient was a 48-year-old man without any history of interest (nonsmoker and without any dyslipidemia) without any significant medication history except circulatory problems of the lower limbs and without any relevant family history. His chief complaint was vision loss when he came to consult our institution. Exploration revealed that his best corrected visual acuity was 20/20 in both eyes. His initial IOP without treatment in the right eye was 14–16 mmHg, and his initial IOP in his left eye was 16–18 mmHg. Slit-lamp exploration revealed mild sclerosis of the lens, and in Goldman gonioscopy, an open angle was confirmed in four quadrants (Shaffer scale grade 4). No inflammation was observed in the anterior chamber. No pigmented dispersion was detected on the corneal surface or lens or angle. In fundoscopic examination, the patient’s vertical cup disc ratio was 0.6 in the right eye and 0.8 in the left eye. His neuroretinal rim was normal in the right eye, and superior thinning in the left eye was detected without any optic disc drusen. In the patient’s visual field, inferior mild probable nasal scotoma was observed in the right eye and an inferior deep arcuate scotoma defect was seen in the left eye (Fig. 1). Optical coherence tomography (OCT) revealed thinning of the peripapillary nerve fiber layer thickness in the left eye and superior loss of the macular RGC in the left eye (acceptable quality of images, right eye, 58; left eye, 48) (Fig. 2).

**Fig. 3:**
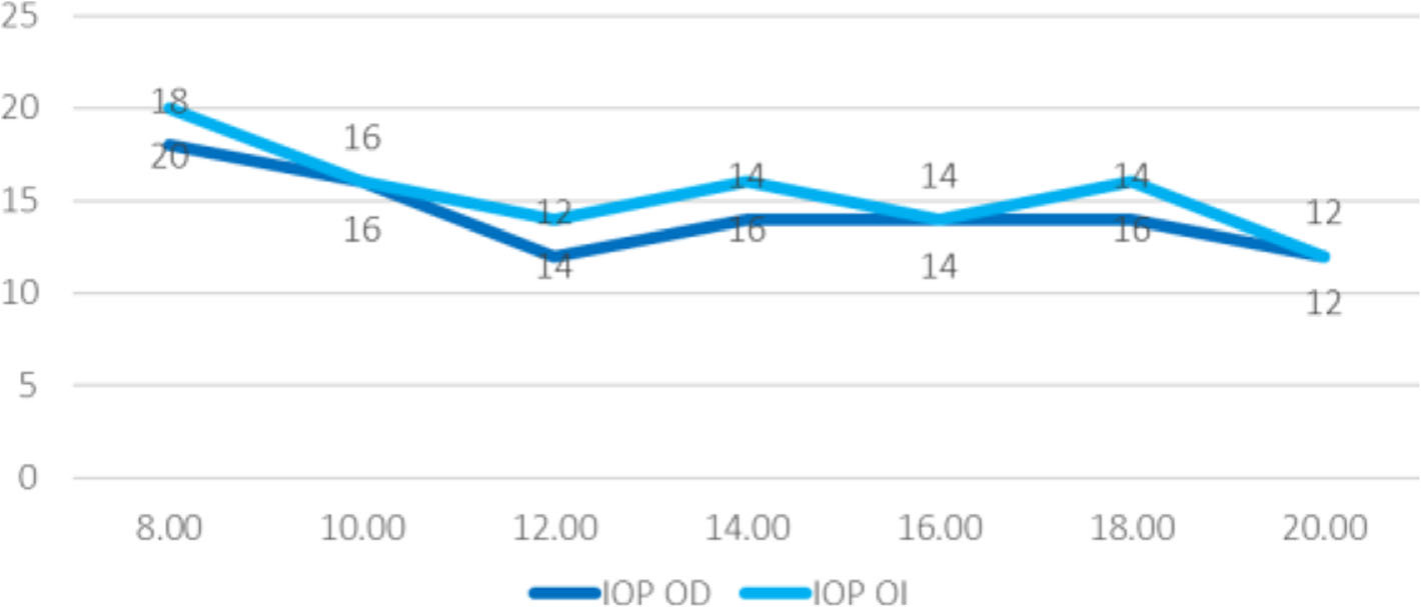
Normal intraocular pressure (IOP) values in diurnal IOP curve without treatment

**Fig. 4:**
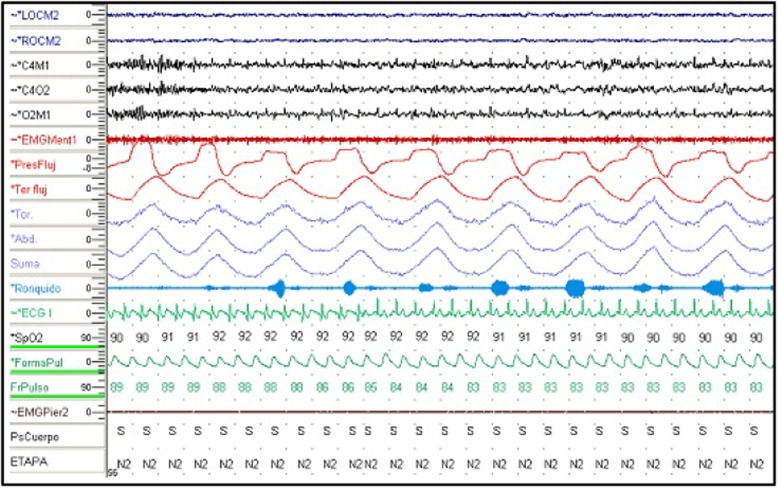
Polysomnography

Normal IOP values were found in the patient’s diurnal IOP curve without treatment (maximum value, 18–20 mmHg), discarding higher IOPs measured out of office (Fig. 3). The patient’s ultrasound pachymetry measurement was 515/520 μm, and he had normal IOP measured with a PASCAL tonometer (no corneal biomechanical underestimation). The result of his polysomnography (Fig. 4) study was normal, excluding sleep apnea syndrome (SAS).

The patient had normal IOP values in his diurnal IOP curve without treatment (maximum value, 18–20 mmHg), discarding higher IOPs measured out of office. The finding of ultrasound pachymetry was 515/520 μm, and the patient had normal IOP measured with a PASCAL tonometer, ruling out probable corneal biomechanical underestimations. The patient had a normal polysomnography study, excluding SAS, and normal macular and peripapillary angio-OCT findings were detected.

The patient’s serial mean BP, especially in the lower limbs (mean value, 125/70 mmHg), ruled out the possibility of systemic hypotension. Thyroid and coagulation abnormalities and autoimmune disease and inflammatory disease were excluded by normal thyroid-stimulating hormone (TSH), T4, prothrombin time (PT), partial thromboplastin time (PTT), bleeding time (BT), and negative anticardiolipin antibody, as well as other immunologic study and normal vascular biopsy. The findings of the patient’s brain magnetic resonance imaging (MRI) were normal, as was his carotid vascular study.

### Primary diagnosis

Moderate medium PAD in the lower limbs was confirmed by echography on the basis of suprapopliteal arterial stenosis (increased systolic rise time and loss of pulsatility). Bilateral lesions were observed in his physical examination, and decreased pulses and some bruits were detected, especially in the left leg, without any resting ankle-brachial index (ABI) abnormalities. His lower BP level of 120/70 mmHg was normal.

## Discussion

A main cause of the disturbed autoregulation of ocular blood flow was the PVD syndrome frequently observed in patients with NTG [6]. The retinal vessels of patients with PVD usually show a higher spatial irregularity and higher stiffness [17, 18], which was not observed in our patient. PVD was known to be a main cause of splinter hemorrhages at the border of the ONH, which may explain why ONH hemorrhages often occur in patients with NTG [19, 20]. In terms of circulation, patients with PVD have an inborn tendency to respond differently to various stimuli, such as cold [21]. Vasoconstriction was the most apparent pathological reaction [6]. In addition, ocular blood flow was correlated with peripheral circulation in patients with PVD, such as in their fingers [22]. A repeated but very mild ocular blood flow decrease, mainly due to disturbed autoregulation and ocular perfusion pressure fluctuation, would lead to an unstable oxygen supply and increased local mitochondrial oxidative stress [23–26]. This process was a recognized pathophysiological mechanism of glaucomatous optic neuropathy. In our patient’s case, we could not detect any related symptomatic PVD, such as finger symptoms, related to cold.

### Vasospasm

Vasospasm may play a key role in ONH damage and may lead to systemic autoregulatory dysfunction in patients with NTG. Previous studies showed that vasospasm created an environmental dysregulation of blood flow, which increased the vulnerability of the ONH to vascular challenges, and this caused perfusion instability, ischemic changes, reperfusion injury, and axonal loss of the ONH [27, 28]. Vasospasm was common and associated with multiple diseases. However, it appeared to be a transient phenomenon that could be reversible by improving retrobulbar hemodynamics in patients with NTG using calcium channel blockers [6]. Although vasospasm is a pathological feature in NTG, we did not observe any related symptoms, which could confirm the pathological cause of vasospasm in our patient’s case.

### Endothelial dysfunction

Previous studies showed that the vascular endothelium regulated the microcirculation through release of vasoactive factors, including the vasodilator nitric oxide (NO) and the vasoconstrictor endothelin-1 (ET-1) [29]. NO released from endothelial cells directly stimulated the surrounding vascular smooth muscle to promote vasodilation [30]. Systemic factors such as hyperlipidemia, atherosclerosis, and hyperglycemia impaired endothelial NO signaling through oxidative stress damage [31]. It was known that NO activity contributed to ocular autoregulation and could protect the endothelium and nerve fiber layer against pathologic stresses implicated in glaucoma [30]. Opposing the vasodilatory properties of NO was ET-1, the most important and potent vascular constricting factor [32]. A number of studies demonstrated the increased plasma ET-1 levels in patients with NTG [33, 34]. *In vitro* and animal studies showed that ET-1 exerted its vasoconstrictor effects mainly on the microvessels in the retina [35], which could reduce the blood supply to the optic nerve [36]. Buckley *et al.* identified that dysfunction of systemic vascular endothelial cells caused decreased responsiveness to ET-1 stimulation in patients with NTG [37]. Endothelial dysfunction is likely related to NTG, and the endothelial dysfunction may be primary or secondary to vascular diseases, including vasospasm and atherosclerosis, in its contribution to NTG pathology.

Endothelial dysfunction plays a key role in atherosclerotic disease. Several methods have been reported to be useful for evaluating endothelial dysfunction. Igari *et al.* demonstrated that the endothelial function measured by peripheral arterial tonometry significantly correlated with the ABI in patients with PAD, and they investigated endothelial dysfunction in patients with PAD [37]. Because the diagnosis of PAD was confirmed in our patient with NTG, we believe that endothelial dysfunction secondary to PAD could be a probable risk factor for and pathological cause of optic nerve damage in our patient’s case, although this probable effect should be confirmed in future studies.

After obtaining normal IOP values in diurnal IOP after discarding higher IOPs measured out of office, corneal biomechanical underestimation was excluded by ultrasound pachymetry measuring 515/520 μm and normal IOP measured with a PASCAL tonometer. Our patient’s normal polysomnography study finding ruled out SAS as a major risk factor related to NTG.

**Fig. 5:**
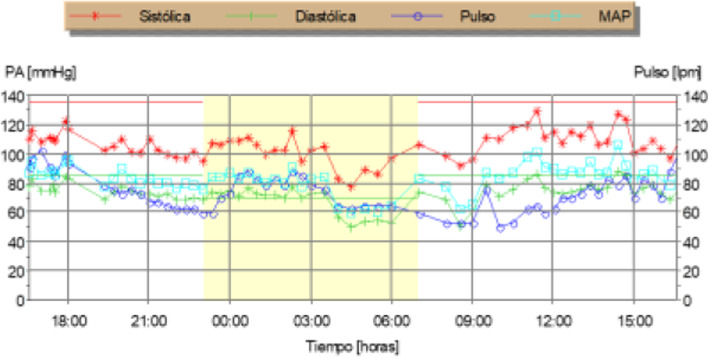
Normal serial blood pressure (BP) during a cycle of 24 hours mean arterial pressure monitoring. Red dots = systolic BP); green dots = diastolic BP; purple dots = pulse; blue dots = mean arterial pressure

**Fig. 6:**
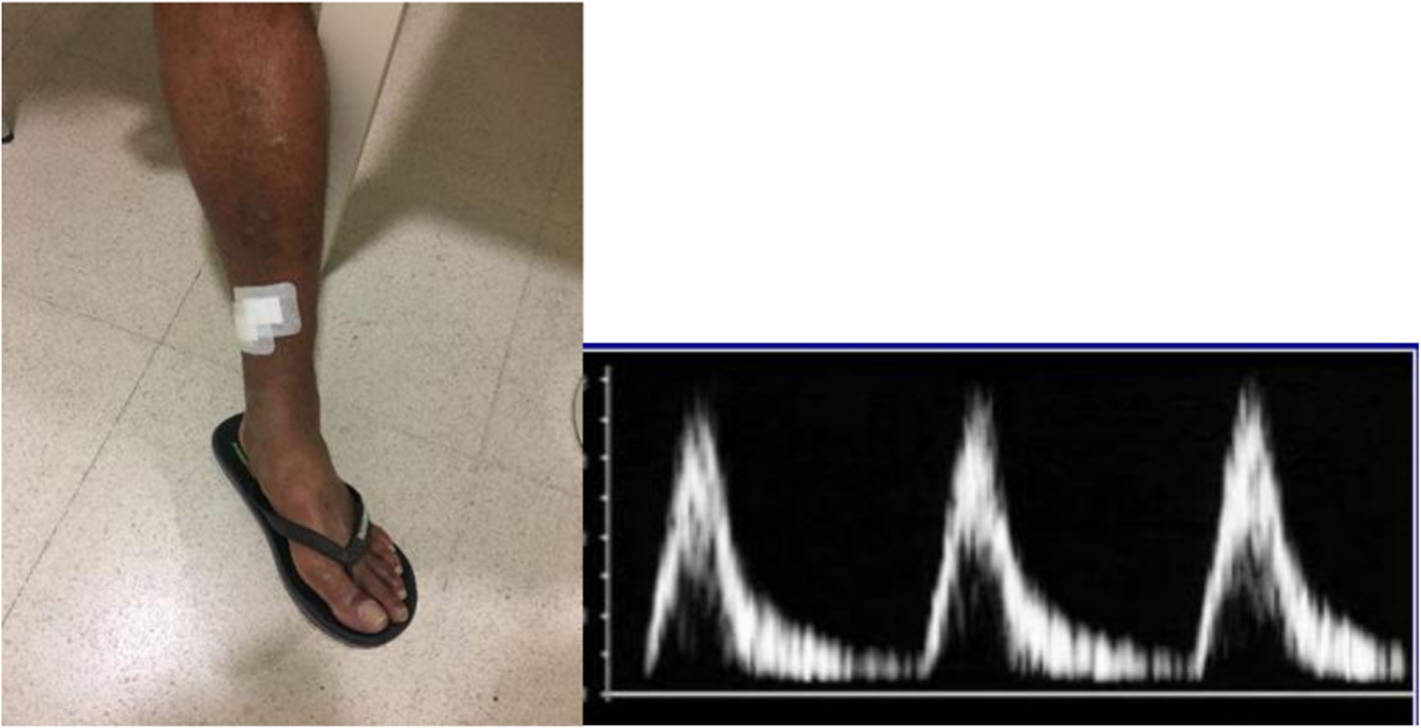
Echography of lower limbs (suprapopliteal arterial stenosis; increased systolic rise time and loss of pulsatility) and anterior and lateral ulcers of lower limb

Our patient’s normal serial BP ruled out the possibility of systemic hypotension (Fig. 5). Thyroid and coagulation abnormalities were excluded by normal TSH, T4, PT, PTT, BT, and anticardiolipin antibody. The findings of our patient’s brain MRI and carotid vascular study were normal. PAD in the lower limbs was confirmed by echography because of lower limb ulcers (Fig. 6).

An association of PAD with glaucoma has been demonstrated in an Asian population, independent of cardiovascular risk factors. These findings provide further support for the concept that vascular processes and mechanisms that are pressure independent are associated with glaucomatous optic neuropathy [38].

In our patient’s case, after ruling out other systemic diseases and vascular abnormalities related to NTG, we found PAD as a probable vascular abnormality related to NTG that causes endothelial dysfunction related to NTG based on Igari *et al.*’s study.

Thus, further research is needed to determine the relevance of these results to the general population, to clarify the temporal nature of the association, and to determine the relationship of PAD with different glaucoma subtypes (primary open-angle glaucoma, primary angle-closure glaucoma, and secondary glaucoma).

## Conclusion

Peripheral vascular disease seems to be associated with glaucoma populations independent of other cardiovascular risk factors, which could be associated with NTG, as reported in our patient’s case, for the first time, to our knowledge, and should be considered as another vascular abnormality related to NTG.

## Abbreviations

ABI: Ankle-brachial index
BP: Blood pressure
BT: Bleeding time
ET-1: Endothelin-1
IOP: Intraocular pressure
MRI: Magnetic resonance imaging
NO: Nitric oxide
NTG: Normal-tension glaucoma
OCT: Optical coherence tomography
ONH: Optic nerve head
PAD: Peripheral arterial disease
PT: Prothrombin time
PTT: Partial thromboplastin time
PVD: Primary vascular dysregulation
RGC: Retinal ganglion cell
SAS: Sleep apnea syndrome
TSH: Thyroid-stimulating hormone
